# Vitamin D improves hepatic steatosis in NAFLD *via* regulation of fatty acid uptake and β-oxidation

**DOI:** 10.3389/fendo.2023.1138078

**Published:** 2023-03-22

**Authors:** Tingwan Du, Lian Xiang, Jingjing Zhang, Chunmei Yang, Wenxin Zhao, Jialu Li, Yong Zhou, Ling Ma

**Affiliations:** ^1^ Department of Nutrition and Food Hygiene, School of Public Health, Southwest Medical University, Luzhou, China; ^2^ Department of Clinical Nutrition, The Affiliated Hospital of Southwest Medical University, Luzhou, China; ^3^ Health Management Center, The Affiliated Hospital of Southwest Medical University, Luzhou, China; ^4^ Department of Medical Cell Biology and Genetics, School of Basic Medical Science, Southwest Medical University, Luzhou, China; ^5^ Environmental Health Effects and Risk Assessment Key Laboratory of Luzhou, School of Public Health, Southwest Medical University, Luzhou, China

**Keywords:** non-alcoholic fatty liver disease, hepatic steatosis, vitamin D, PPARα, lipid metabolism

## Abstract

**Introduction:**

The study aimed to explore the association of serum 25(OH)D_3_ and hepatic steatosis in non-alcoholic fatty liver disease (NAFLD) patients and to determine whether the effect of vitamin D (VD) is mediated by activation of the peroxisome proliferator-activated receptor α (PPARα) pathway.

**Methods:**

The study contained a case-control study, *in vivo* and *in vitro* experiments. A case-control study was conducted to compare serum parameters between NAFLD patients and controls and to evaluate the association of 25(OH)D_3_ and NAFLD. *In vivo* study, male Wistar rats were randomly divided into control and model groups, fed a standard chow diet and a high-fat diet (HFD), respectively, for 7 weeks to generate an NAFLD model. Then, the rats were treated with VD and a PPARα antagonist (MK886) for 7 weeks. Tissue and serum were collected and assessed by biochemical assays, morphological analysis, histological analysis, and western blot analysis. *In vitro*, HepG2 cells were incubated with oleic acid (OA) to induce steatosis, which was evaluated by staining. HepG2 cells were pretreated with MK886 followed by calcitriol treatment, and differences in lipid metabolism-related proteins were detected by western blot.

**Results:**

NAFLD patients were characterized by impaired liver function, dyslipidemia, and insulin resistance. Serum 25(OH)D_3_ was negatively associated with alanine aminotransferase (ALT) in NAFLD. VD deficiency was a risk factor for patients with no advanced fibrosis. Adequate VD status (25(OH)D_3_ >20 ng/mL) had a protective effect in patients after adjustment for confounding variables. NAFLD rats showed hyperlipidemia with severe hepatic steatosis, systematic inflammation, and lower serum 25(OH)D_3_. VD treatment ameliorated hepatic steatosis both in NAFLD rats and OA-induced HepG2 cells. Further, MK886 inhibited the anti-steatosis effect of VD.

**Conclusion:**

The study revealed that an adequate VD level may act as a protective factor in NAFLD and that VD may alleviate hepatic steatosis *via* the PPARα signaling pathway.

## 1 Introduction

With the worldwide spread of a sedentary lifestyle and the Western diet, non-alcoholic fatty liver disease (NAFLD)—characterized by excessive fat accumulation in hepatocytes caused by factors other than alcohol abuse—is becoming a global health problem with approximately 30% of the world suffering from the disease (1). As NAFLD progresses, its pathology ranges from non-alcoholic fatty liver (NAFL) to non-alcoholic steatohepatitis (NASH), fibrosis, and ultimately to hepatocellular carcinoma (HCC) (2). The “multiple-hit” hypothesis for the pathogenesis of NAFLD has gradually been replaced by the “two-hit” hypothesis. Thus, NAFLD is no longer considered a single process but instead a disease caused by adipose tissue (AT) lipolysis (3, 4), gut microbiota (5, 6), and liver dysfunction (4). Moreover, NAFLD is affected by dietary factors, genetic factors, and other factors (7, 8). The etiology of NAFLD is still unclear. Although a specific medicine for NAFLD is still in development, weight loss and lifestyle modification are considered efficient treatments (9).

The liver is the largest solid organ and plays a crucial role in maintaining energy homeostasis through various nutrients, including lipids. For healthy individuals, a balance exists between fatty acid influx and disposal in the liver. The source of influx can be divided into fatty acid uptake (AT lipolysis and dietary fat) and *de novo* synthesis. Intrahepatic mitochondrial β-oxidation and secretion of very low-density lipoprotein (VLDL) are the main processes of fatty acid degeneration (10, 11). Hepatic steatosis, caused by a disrupted lipid metabolism balance, is defined by the presence of intracellular triglyceride (TG) accumulation (>5% of hepatocytes), which is the first stage of NAFLD pathogenesis. Dyslipidemia, characterized by elevated TG, total cholesterol (TC), VLDL, and low-density lipoprotein cholesterol (LDL-C) as well as reduced high-density lipoprotein cholesterol (HDL-C) caused by hepatic lipid metabolic disorder, is a frequent feature of NAFLD patients (12–14). Lipid homeostasis is maintained by the regulation of hormones, nuclear receptors, and transcription factors. Peroxisome proliferator-activated receptor α (PPARα), as a ligand-activated nuclear receptor, is highly expressed in high fatty acid oxidative (FAO) tissues and acts as a nutritional sensor. PPARα directly regulates the transcription of target genes by forming a heterodimer with the retinol X receptor (RXR) and binding to the target gene promoter or peroxisome proliferative response element (PPRE) sequence through the deoxyribonucleic acid (DNA)-binding domain (15–17). In addition, PPARα represses related gene expression in trans-acting, cis-acting, and indirect manners (18). PPARα ligands include lipophilic endogenous substances with nuclear permeation, mainly derived from nutrients and exogenous chemicals. The beneficial effects of PPARα on NASH have been demonstrated *in vivo* as a therapeutic target for several medicines (19–21). Therefore, potential agonists of PPARα are still being explored (22–24).

Free fatty acids (FFAs) are transferred from plasma into cells by the membrane-associated transporter, fatty acid translocase (FAT/CD36), which is positively regulated by PPARα ligands (25). Under physiological conditions, CD36 expression is low in hepatocytes, but CD36 is highly induced with lipid overload and is activated by transcription factors (26). Once FFAs enter hepatocytes, they are bound and transported by fatty acid binding protein 1 (FABP1), and functional PPREs identified within the FABP1 promoter are regulated by PPARα based on nutritional conditions through protein–protein interactions (15, 27–29). Carnitine palmitoyltransferase 1A (CPT1A), which transports FFAs into mitochondria, is localized in the outer mitochondrial membrane and acts as a β-oxidation rate-limiting enzyme regulated by PPARα (15, 30). As the regulator of lipid metabolism, PPARα is an attractive target in NAFLD therapy. The MK886 compound has been identified as a selective inhibitor of PPARα as it prevents conformational change for active complex formation, and it is commonly used in exploring therapeutic targets for NAFLD treatment (31–35).

Vitamin D (VD), known for its role in regulating calcium and phosphorus metabolism, is one of the common fat-soluble vitamins. Recent studies have shown that VD is often associated with several metabolic diseases, such as diabetes (36) and obesity (37). Our previous study showed that 1,25(OH)_2_D_3_ affects the growth of lipid droplets (LDs) by decreasing the expression of TG synthesis genes and increasing the expression of TG lipolysis genes in 3T3-L1 adipocytes (38). Excessive FFAs from lipolysis are transported from AT to the liver (39). A previous cross-sectional study based on the National Health and Nutrition Examination Survey (NHANES) from 2003 to 2004 and from 2005 to 2006 in the United States reported that serum VD level is negatively correlated with NAFLD risk (40). However, a previous cross-sectional study that randomly recruited 789 adult individuals from Portugal has reported that there is no correlation between serum VD and hepatic steatosis (41). A previous meta-analysis involving eight randomized controlled trials (RCTs) verified that VD supplementation has a protective effect on insulin resistance (IR) and decreases serum alanine aminotransferase (ALT) in NAFLD patients (42). A prospective cohort study in Korea reported that maintaining an adequate 25(OH)D_3_ level (≥30 ng/mL) is associated with decreased incidence of NAFLD (43). Using an animal model, Li et al. (39) reported that a long-term VD deficiency diet leads to significant NASH. Moreover, Jahn et al. (44) reported that a preventive VD treatment significantly reduces lipogenic gene expression in mouse hepatic tissue.

We previously reported that 1,25(OH)_2_D_3_ inhibits LDs, promotes LD decomposition, reduces LD volume, and inhibits lipogenesis in 3T3-L1 cells through the PPARα signaling pathway (38). However, the therapeutic effect and underlying mechanism of VD in hepatic lipid metabolism still need to be further explored. Therefore, we investigated serum 25(OH)D_3_ and related clinical factors in a case-control study. We hypothesized that VD may attenuate hepatic steatosis *via* the PPARα pathway and that these effects may be inhibited by MK886 in a NAFLD rat model and oleic acid (OA)-induced HepG2 cells.

## 2 Materials and methods

### 2.1 Participants and study design

The present study included a case-control study. Participants were voluntarily recruited at the Health Management Center of the Affiliated Hospital of Southwest Medical University in China between April 2019 and September 2022. The diagnosis of NAFLD was based on the Guidelines for the Prevention and Treatment of Non-alcoholic Fatty Liver Disease (2018 update) (45) as follows: age between 18 and 70 years old; and diagnosed as fatty liver disease by ultrasound scanning with alcohol consumption ≤210 g/week in men and ≤140 g/week in women in the last 12 months.

Alcohol consumption was calculated as previously reported (46). Participants were excluded if they conformed to one of the following criteria: pregnancy or lactating women; kidney diseases; cardiovascular diseases; and taking drugs that may affect hepatic or VD metabolism (47). Based on previous studies, the prevalence of VD insufficiency (<20 ng/mL) in Chinese adults was 63.2% with an odds ratio (OR) of 2.067 (48, 49). The significant level (α) was 5%, and the power of test (1-β) was 80%. After calculation using PASS (PASS 15.0, NCSS, USA), the minimum sample size of NAFLD cases required was estimated to be 367. The present study ultimately included 381 diagnostic NAFLD patients and 350 age- and gender-matched controls. The present study was approved by the Ethic Research Committee of the Affiliated Hospital of Southwest Medical University, and each participant agreed and signed informed consent.

### 2.2 Anthropometric examination and blood sampling

After a 12-h overnight fast and 48-h alcohol abstinence, all participants underwent a physical examination in the morning. The height, weight, waist circumstance (WC), systolic pressure (SBP), and diastolic blood pressure (DBP) of the participants were measured and recorded by a professional nurse. Fasting venous blood was collected and centrifuged to obtain serum for detection of the following parameters using an automatic biochemical analyzer (AU480, Olympus, Japan): aspartate aminotransferase (AST), ALT, TG, TC, HDL-C, LDL-C, γ-glutamine transferase (GGT), alkaline phosphatase (ALP), albumin (ALB), plasma glucose (GLU), fasting insulin (INS), and blood platelet (PLT). Serum 25(OH)D_3_ was detected by a chemiluminescence immunoassay (CLIA) (VD-T, Mindray, China). Homeostatic Model Assessment for Insulin Resistance (HOMA-IR) was estimated as previously described (50). Body mass index (BMI) was calculated using the following equation: BMI (kg/m^2^) = body weight (kg)/height^2^ (m). Participants with a BMI greater than 25 but less than 30 were defined as overweight, and participants with a BMI greater than 30 were defined as obese (51). According to the Vitamin D Deficiency Screening Method of China in 2020, VD deficiency was defined as circulating 25(OH)D_3_ less than 12 ng/mL, and VD insufficiency was defined as serum 25(OH)D_3_ levels between 12 and 20 ng/mL (52).

### 2.3 Identification of hepatic fibrosis and hepatic steatosis

NAFLD fibrosis score (NFS), Fibrosis-4 (FIB-4) index, and fatty liver index (FLI) were used to identify the presence of fibrosis and hepatic steatosis. NFS was calculated using the following equation: NFS = -1.675 + 0.037 × age (years) + 0.094 × BMI (kg/m^2^) + 1.13 × IR/diabetes (yes=1, no=0) + 0.99 × AST/ALT – 0.013 × PLT – 0.66×ALB (g/dL). The FIB-4 index was calculated as follows: FIB-4 = age × AST / (PLT × √ALT. The FLI was calculated using the following equation: FLI = (e^0.953×ln(TG) + 0.139×BMI + 0.718 × ln(γGT) + 0.053 × WC – 15.745^)/(1 + e^0.953×ln(TG) + 0.139×BMI + 0.718 × ln(γGT) + 0.053 × WC – 15.745^) × 100. The cases were individually divided into the following two groups based on the NFS and FIB-4: no advanced fibrosis (NFS<-1.455 or FIB-4<1.30) and fibrosis (NFS≥-1.455 or FIB-4≥1.30) (53, 54). Based on an FLI cutoff of 60, the cases were divided into the following two groups: no fatty liver (FLI <60) and fatty liver (FLI≥60). (55).

### 2.4 Animal models and experimental protocols

Five-week-old male Wistar rats (140–150 g) were purchased from Chengdu Dossy Experimental Animal Co., Ltd. (Chengdu, Sichuan, China). Rats were housed in controlled environment (temperature of 23 ± 1°C and humidity of 50 ± 10%) with a 12-h light/dark cycle and free access to food and drink.

First, VD treatment and the underlying mechanisms of VD in NAFLD pathogenesis were explored. In total, 48 rats were stratified by body weight and randomly divided into the following two groups: CON group (n=16), which received 14 weeks of standard chow diet, and high-fat diet (HFD) group (n=32), which received 14 weeks of HFD throughout the experiment (**Supplementary Table 1**). At Week 7, eight rats were killed in each group to determine successful establishment of the NAFLD model. For the next 7 weeks, the CON group (n=8) received a standard chow diet. The HFD group was stratified by body weight and randomly divided into the following three groups to receive intervention for 7 weeks: HFD group (n=8), HFD + VD group (n=8), and HFD + Oil group (n=8). In the HFD + VD group, the concentration of cholecalciferol (VD; 47763, Sigma, Germany) dissolved in corn oil (C116025, Aladdin, China) was 2.5 μg/ml, and the gavage dose was 12.5 μg/kg as in previous studies (56–59). In the HFD + Oil group, the gavage dose of corn oil was based on the volume used to administer VD.

Second, we investigated whether the improvement of hepatic metabolism by VD is associated with the PPARα pathway. In total, 24 rats were fed a HFD for 14 weeks. At Week 7, the rats were randomly divided into the following three groups to receive intervention: HFD + DMSO group (n=8), HFD + MK886 group (n=8), and HFD + MK886 + VD group (n=8). MK886 (No. 21753, Cayman, USA) was dissolved in dimethyl sulfoxide (DMSO) (D8370-100 ml, Solarbio, China) with carboxymethylcellulose sodium (CMC-Na) (C8621, Solarbio, China) at a concentration of 0.2 mg/ml, and the intraperitoneal injection dose was 1 mg/kg per day (60). During the experimental period, the body weights were measured and recorded every week. In addition, the food was measured, and additional food was added each day at 4:00 PM to allow free access to food and drink.

At the end of Week 14, all rats were anesthetized with pentobarbital sodium (45 mg/kg body weight). Serum samples were isolated after centrifugation for subsequent analyses. The livers of each animal were removed and weighed immediately after excision, and they were stored at -80°C until examination. The hepatosomatic index was calculated as follows: liver index = liver weight (g)/body weight (g) × 100%. Portions of liver tissue were fixed in paraformaldehyde for conventional staining. All methods in the animal study were performed following the 1996 National Institute of Health Guide for the Care and Use of Laboratory Animals. All experimental protocols were approved by the Ethics Committee of Southwest Medical University in accordance with the ARRIVE guidelines (Approval No. 2020612; approval date of August 17, 2020).

### 2.5 Liver pathological evaluation

Hematoxylin and eosin (H&E) staining (BA-4097, BaSO, China) was performed according to standard procedure to evaluate pathological injury. Liver tissue was fixed in 4% paraformaldehyde for 24 h, embedded in paraffin, sectioned, and stained with H&E. Four rats were selected for each group, and one image was acquired for each rat to obtain the NAS, which was performed by a pathologist according to three components, namely, hepatic steatosis, lobular inflammation, and ballooning degeneration, as previously described (61). Oil red O (ORO) staining (BMB2117, Bomeibio, China) was used to determine intracellular lipids. Fresh livers were fixed using 4% paraformaldehyde (BL539A, Biosharp, China), dehydrated, embedded in paraffin, and sectioned using an automatic microtome. The sections were immersed in 60% isopropyl alcohol, stained with ORO for 10 min, immersed in 60% isopropyl alcohol, washed with distilled water, stained with hematoxylin (ZH202509, Servicebio, China), and immersed in hydrochloric acid for 3 min. The sections were then sealed with glycerin gelatin. Images were acquired using a microscope (BA200Digital, Motic, China). The integrated optical density (IOD) and area of all images were measured using Image-Pro Plus 6.0 (USA), and the percentage of LDs was calculated.

### 2.6 Metabolic biochemical analysis

The serum levels of AST, ALT, TG, TC, HDL, LDL, and GLU were detected by an automatic biochemical analyzer (AU480, Olympus, Japan). Serum cytokines, including tumor necrosis factor-α (TNF-α) (ERC102a, NeoBioscience, China), monocyte chemotactic protein-1 (MCP-1) (ERC113, NeoBioscience, China), and interleukin- 6 (IL-6) (ERC003, NeoBioscience, China), as well as 25(OH)D_3_ (E-EL-0015c, Elabscience, China) were measured by enzyme-linked immunosorbent assay (ELISA) kits according to the manufacturer’s instructions. Liver tissues were homogenized in a working solution and centrifuged at 12000 × g for 20 min. The TG contents (A110-1-1, Jiancheng Bio, China) and FFA contents (A042-2-1, Jiancheng Bio, China) of the liver tissue were detected according to manufacturer’s instructions. The lipid level was normalized with the respective protein concentration.

### 2.7 Cell culture and induced fatty liver cells

HepG2 hepatocyte cells were cultured at 37°C, 5% CO_2_, and constant pH (7.2-7.4) in Dulbecco’s Modified Eagle Medium (DMEM; C11995500BT, Gibco, USA) supplemented with 10% (v/v) fetal bovine serum (FBS; SA211.02, Cellmax, Australia), 100 U/mL penicillin, and 100 μg/mL streptomycin (C0222, Beyotime, China). OA (O7501, Sigma, Germany) was conjugated to 10% (v/v) bovine serum albumin (BSA) (ST025, Beyotime, China). To induce hepatic steatosis, HepG2 cells were exposed to 0.5 mM OA for 24 h to induce lipid accumulation (62–64). Cells were then washed, cultured in fresh complete medium, and treated with different drugs for further analysis.

### 2.8 Cell viability assay

The effects of calcitriol (HY-10002, MCE, USA) and MK886 on cell viability were measured using the cell counting kit-8 (CCK8) assay (CT01, Cellcook, China). HepG2 cells were seeded in 96-well plates at a density of 1 × 10^4^ cells/well. After 24 h of attachment, cells were pretreated with 0.5 mM OA for 24 h followed by treatment with different concentrations of calcitriol (0, 25, 50, 100, and 200 nM; dissolved in ethanol, HY-10002, MCE) at 37°C for 24 h. MK886 (0.1, 1, 10, and 100 μM, dissolved in DMSO) was added for 0.5 h, 1 h, and 2 h before calcitriol treatment. The final concentration of DMSO (D4540, Sigma, USA) and ethanol in culture medium did not exceed 0.1% to minimize toxicity. Next, 10 μL of CCK8 reagent (CT01A, Cellcook, China) dissolved in 90 μL of DMEM was added to each well followed by incubation for 2 h. Finally, the absorbance of each well was detected at 450 nm using a microplate reader (Synergy H1, BioTek, USA).

### 2.9 Quantification of intracellular lipid accumulation

To visualize intracellular LDs, HepG2 cells were washed twice with phosphate buffer saline (PBS) (P1003-2 L, Solarbio, China) and fixed in 4% paraformaldehyde for 30 min. Cells were washed with detergent for 30 s and then incubated with ORO working solution (C0158S, Beyotime, China) for 30 min. Cells were decolorized with detergent for 30 s and washed with PBS. Finally, images were acquired using a microscope (CKX53, Olympus, Japan). To determine intracellular TG level, HepG2 cells were seeded into 6-well plates and allowed to attach overnight. Cells were then treated with OA (0.5 mM) and calcitriol (0-200 nM) as described above. The contents of TG, TC, and protein in the lysate were determined by a TG kit (A110-1-1, Jiancheng Bio, China), TC kit (A111-1-1, Jiancheng Bio, China), and bicinchoninic acid (BCA) kit (PC0020, Solarbio, China), respectively. The ratio of lipid level to protein concentration was calculated to determine the relative lipid level.

### 2.10 Western blot analysis

Total protein was extracted from the liver tissue and HepG2 cells, and the total protein concentration was determined using the BCA method. Proteins were separated by 10% sodium dodecyl sulfate–polyacrylamide gel electrophoresis (SDS-PAGE) (P1200, Solarbio, China) and transferred to polyvinylidene fluoride (PVDF) membranes (ISEQ00010, Millipore, USA). The membranes were blocked with 5% nonfat milk (P0216-300g, Solarbio, China) containing 0.1% Tween^®^ 20 (T8220, Solarbio, China). After washing with 0.1% Tris-buffered saline with Tween^®^ 20 (TBST), the membranes were incubated with specific antibodies overnight. After being washed in TBST, the membranes were incubated with anti-rabbit immunoglobulin G (IgG) and horseradish peroxidase (HRP)-linked antibody diluted at 1:2000 (7074S, CST, USA) for 1-h. The protein expression was detected by a GelView system (6000plus, BLT, China), and immunoreactive bands were analyzed by ImageJ (ImageJ 1.45s, USA). The specific antibodies were against PPARα (ab24509, 1:500, Abcam, China), CPT1A (bs-2470r, 1:2000, Bioss, China), CD36 (bs-1100r, 1:2000, Bioss, China), and FABP1 (ab153924, 1:2000, Abcam, UK). β-actin (4970T, 1:3000, CST, USA) was used as an internal control.

### 2.11 Statistical analysis

All statistical analyses were performed using SPSS 26.0 (SPSS Inc., USA) and GraphPad 8.0 (GraphPad Software, USA). The Shapiro–Wilk test was used to assess the normality of continuous variable distribution. Data are expressed as the mean ± standard error of mean (SEM) or median (interquartile range). Student’s t-tests or the Mann–Whitney test and χ^2^ test were used to assess differences between groups. Group data were analyzed using one-way analysis of variance (ANOVA) with least significant difference (LSD) or Games–Howell tests. Spearman’s correlation was used to assess the association of 25(OH)D_3_ with serum indicators of NAFLD. NFS, FIB-4, and FLI levels were categorized into two levels according to their cutoff values. Binary logistic regression was used to assess the association between serum parameters and NAFLD. Multiple logistic regression was performed to explore the association between 25(OH)D_3_ status and NAFLD after adjustment for confounding factors. P<0.05 (two-sided) was considered to be significant.

## 3 Results

### 3.1 Clinical and demographic characteristics of participants

The clinical and demographic characteristics of participants are listed in **Tables 1**, **2**. Compared to controls, NAFLD patients had higher BMI, WC, SBP, DBP, ALT, AST, ALP, GGT, INS, GLU, HOMA-IR, TC, TG, and LDL but lower HDL-C. The trend persisted in NAFLD patients and controls after gender stratification (all P<0.05). The prevalence of hypertension (22.0% *vs*. 5.1%), diabetes (7.3% *vs*. 1.7%), overweight (54.9% *vs*. 16.0%), and obesity (12.9% *vs*. 0.6%) was higher in NAFLD patients than in controls (all P<0.05, **Table 2**). The prevalence of hypertension (males: 19.3% *vs*. 5.6% and female: 24.9% *vs*. 4.8%), diabetes (male: 6.8% *vs*. 0.6% and female: 7.9% *vs*. 2.7%), overweight (male: 57.3% *vs*. 22.7% and female: 52.4% *vs*. 10.2%), and obesity (male: 18.7% *vs*. 0.6% and female: 7.0% *vs*. 0.6%) were higher in NAFLD patients than in controls when stratified by gender.

**Table 1:** Clinical and biological characteristics of NAFLD and controls.

Variable	NAFLD			Control			*p^1^*	*p^2^*	*p^3^*
	Male (n=192)	Female (n=189)	Total (n=381)	Male (n=163)	Female (n=187)	Total (n=350)			
Age (year)	45.00 (33.00-55.00)	53.00 (48.00-59.00)	50.00 (37.50-56.00)	44.00 (34.00-52.00)	51.00 (45.00-57.00)	49.00 (38.00-55.00)	0.768	0.054	0.245
BMI (kg/m^2^)	26.98 (25.16-29.13)	25.59 (23.64-27.55)	26.30 (24.36-28.41)	23.32 (21.85-24.93)	21.84 (20.32-23.34)	22.58 (20.97-24.32)	<0.001	<0.001	<0.001
WC (cm)	93.00 (87.00-99.00)	84.00 (77.00-89.00)	88.00 (82.00-95.00)	83.00 (78.00-87.00)	74.00 (69.00-79.00)	78.00 (72.00-84.00)	<0.001	<0.001	<0.001
SBP (mmHg)	127.00 (117.00-136.00)	126.00 (114.50-136.00)	127.00 (116.00-136.00)	120.00(111.00-128.00)	116.00(106.00-125.00)	118.00(108.00-126.00)	<0.001	<0.001	<0.001
DBP (mmHg)	79.00 (72.00-86.00)	75.00 (67.00-81.50)	76.50 (69.25-84.00)	73.00 (66.00-80.00)	69.00 (63.75-75.25)	71.00 (65.00-77.00)	<0.001	<0.001	<0.001
ALT (U/L)	33.70 (24.00-53.63)	24.10 (17.8-32.85)	28.40 (19.40-41.15)	23.50 (18.20-31.30)	17.70 (12.90-22.80)	20.05 (14.85-26.80)	<0.001	<0.001	<0.001
AST (U/L)	26.20 (21.83-31.78)	22.20 (19.10-27.60)	24.20 (20.20-29.90)	22.20 (19.80-27.00)	21.20 (18.50-25.20)	21.70 (19.10-25.93)	<0.001	0.046	<0.001
GGT (U/L)	38.60 (26.28-59.33)	20.80 (15.85-30.10)	28.10 (18.80-45.15)	22.70 (16.80-33.60)	13.60 (10.70-18.50)	17.40 (12.20-26.80)	<0.001	<0.001	<0.001
ALP (U/L)	75.15 (62.33-87.63)	73.90 (59.75-90.90)	74.70 (61.40-88.80)	72.10 (59.10-84.20)	64.30 (53.60-81.30)	67.70 (56.78-83.60)	0.057	<0.001	<0.001
ALB (g/L)	47.45 ± 2.46	45.77 ± 2.12	46.62 ± 2.45	47.24 ± 2.53	45.44 ± 2.46	46.28 ± 2.65	0.440	0.164	0.075
INS (μIU/mL)	12.17 (8.51-16.85)	12.31 (8.84-18.52)	12.28 (8.61-17.04)	6.45 (4.66-9.16)	6.79 (4.91-10.16)	6.48 (4.85-9.16)	<0.001	<0.001	<0.001
GLU (mmol/L)	5.25 (4.85-6.02)	5.31 (4.95-5.71)	5.30 (4.91-5.80)	4.92 (4.59-5.20)	4.86 (4.63-5.21)	4.89 (4.60-5.21)	<0.001	<0.001	<0.001
HOMA-IR	3.00 (2.13-4.08)	2.90 (2.15-3.95)	2.90 (2.15-3.95)	1.45 (1.00-2.00)	1.50 (1.00-2.18)	1.50 (1.00-2.08)	<0.001	<0.001	<0.001
TC (mmol/L)	5.10 ± 1.09	5.24 ± 1.11	5.17 ± 1.10	4.75 ± 0.83	5.17 ± 1.02	4.97 ± 0.95	0.001	0.553	0.012
TG (mmol/L)	2.19 (1.47-3.41)	1.70 (1.35-2.60)	1.94 (1.40-2.94)	1.25 (0.88-1.78)	1.09 (0.80-1.44)	1.15 (0.82-1.60)	<0.001	<0.001	<0.001
HDL (mmol/L)	1.07 ± 0.22	1.28 ± 0.30	1.17 ± 0.29	1.35 ± 0.30	1.65 ± 0.35	1.51 ± 0.36	<0.001	<0.001	<0.001
LDL (mmol/L)	3.09 ± 1.01	3.30 ± 0.95	3.20 ± 0.98	2.98 ± 0.80	3.06 ± 0.91	3.02 ± 0.86	0.252	0.013	0.012
25(OH)D_3_ (ng/mL)	16.92 (13.48-24.71)	16.64 (12.45-20.80)	16.78 (12.90-22.63)	19.44 (15.20-25.63)	16.90 (12.30-22.38)	17.72 (13.81-23.35)	0.073	0.485	0.111

NAFLD non-alcoholic fatty liver disease; BMI, body mass index; WC, Waist circumference; SBP, Systolic blood pressure; DBP, Diastolic blood pressure; ALT, Alanine Transaminase; AST, Aspartate Aminotransferase; GGT, γ-glutamyl transpeptidase; ALP, alkaline phosphatase; ALB, Albumin; INS, Insulin; GLU, glucose; HOMA-IR, Homeostasis model assessment insulin resistance; TC, total cholesterol; TG, triglyceride; HDL, High-density lipoprotein; LDL, Low-density lipoprotein.

^1^NAFLD male vs. Control male. ^2^NAFLD female vs. Control female. ^3^NAFLD vs. Control.

**Table 2:** Description of counting data in NAFLD and controls.

Variable	NAFLD			Control			*p^1^*	*p^2^*	*p^3^*
	Male (n=192)	Female (n=189)	Total (n=381)	Male (n=163)	Female (n=187)	Total (n=350)			
Hypertension n (%)	37 (19.3)	47 (24.9)	84 (22.0)	9 (5.6)	9 (4.8)	18 (5.1)	<0.001	<0.001	<0.001
Diabetes n (%)	13 (6.8)	15 (7.9)	28 (7.3)	1 (0.6)	5 (2.7)	6 (1.7)	0.003	0.023	<0.001
BMI (kg/m^2^)							<0.001	<0.001	<0.001
<25	46 (24.0)	76 (40.6)	122 (32.2)	125 (76.7)	166 (89.2)	291 (83.4)	–	–	–
25-30	110 (57.3)	98 (52.4)	208 (54.9)	37 (22.7)	19 (10.2)	56 (16.0)	–	–	–
≥30	36 (18.7)	13 (7.0)	49 (12.9)	1 (0.6)	1 (0.6)	2 (0.6)	–	–	–
25(OH)D_3_(ng/mL)							0.109	0.556	0.162
<12	21 (14.0)	30 (18.4)	51 (16.3)	12 (8.9)	40 (23.4)	52 (17.0)	–	–	–
12-20	72 (48.0)	88 (54.0)	160 (51.1)	61 (45.2)	69 (40.4)	130 (42.5)	–	–	–
≥20	57 (38.0)	45 (27.6)	102 (32.6)	62 (45.9)	62 (36.2)	124 (40.5)	–	–	–

NAFLD non-alcoholic fatty liver disease; BMI. Body mass index.

^1^NAFLD male vs. Control male. ^2^NAFLD female vs. Control female. ^3^NAFLD vs. Control.

### 3.2 Low VD is a risk factor for NAFLD

As shown in **Table 3**, 25(OH)D_3_ was negatively correlated with ALT and BMI in NAFLD patients, whereas other indicators showed no significant correlation with serum 25(OH)D_3_ in NAFLD patients. The association between VD status and fibrosis severity was further analyzed. There was a positive association between VD insufficiency and liver steatosis (OR=1.959, 95% CI: 1.061–3.616, P=0.030) (**Table 4**). Moreover, VD insufficiency was identified as a risk factor for no advanced fibrosis, which was reflected in the NFS (OR=1.470, 95% CI: 1.047–2.064, P=0.026) and FIB-4 index (OR=1.652, 95% CI: 1.158–2.357, P=0.005) (**Table 4**). Before adjusting for potential confounding factors, a high level of 25(OH)D_3_ (>20 ng/mL) was not associated with NAFLD risk (OR=0.839, 95% CI: 0.526–1.338, P _trend_=0.075). After adjusting for age and gender (OR=0.740, 95% CI: 0.457–1.198, P _trend_=0.035) or age, gender, BMI, INS, and GLU (OR=0.076, 95% CI: 0.006–1.037, P _trend_<0.001), a high level of 25(OH)D_3_ was not associated with NAFLD. When the model was adjusted for age, gender, BMI, INS, GLU, ALT, GGT, and ALP, a significant protective effect was found between a high level of 25(OH)D_3_ (>20 ng/mL) and NAFLD (OR=0.019, 95% CI: 0.001–0.619, P _trend_<0.001) (**Table 5**). Therefore, a high VD level (>20 ng/mL) may be a protective factor against NAFLD.

**Table 3:** Correlation of clinical factors with 25(OH)D_3_ among NAFLD and controls.

Clinical factors	NAFLD		Control		Total	
	r	*p*	r	*p*	r	*p*
25(OH)D_3_ (ng/mL)						
BMI (kg/m^2^)	-0.122	0.031	0.154	0.007	-0.025	0.540
WC (cm)	-0.088	0.124	0.131	0.023	-0.019	0.637
ALT (U/L)	-0.149	0.008	0.112	0.050	-0.016	0.690
AST (U/L)	0.012	0.838	0.129	0.024	0.057	0.156
ALP (U/L)	-0.060	0.288	0.015	0.791	-0.030	0.456
GGT (U/L)	-0.015	0.785	-0.010	0.861	-0.036	0.377
INS (μIU/mL)	-0.024	0.882	0.232	0.236	-0.027	0.823
GLU (mmol/L)	0.085	0.133	0.014	0.812	-0.065	0.108
HOMA-IR	-0.059	0.705	0.213	0.276	-0.048	0.693
TC (mmol/L)	0.004	0.945	-0.029	0.615	-0.021	0.610
TG (mmol/L)	-0.104	0.068	-0.078	0.175	-0.124	0.002
HDL(mmol/L)	0.090	0.112	-0.079	0.166	0.058	0.153
LDL (mmol/L)	-0.054	0.339	0.010	0.858	-0.038	0.348

NAFLD non-alcoholic fatty liver disease; BMI, body mass index; WC, Waist circumference; SBP, Systolic blood pressure; DBP, Diastolic blood pressure; ALT, Alanine Transaminase; AST, Aspartate Aminotransferase; GGT, γ-glutamyl transpeptidase; ALP, alkaline phosphatase; ALB, Albumin; INS, Insulin; GLU, glucose; HOMA-IR, Homeostasis model assessment insulin resistance; TC, total cholesterol; TG, triglyceride; HDL, High-density lipoprotein; LDL, Low-density lipoprotein.

**Table 4:** Association between vitamin D insufficiency and liver fibrosis index of NAFLD patients and controls.

group	VD insufficiency (<20 ng/mL)	Non-VD insufficiency (≥20 ng/mL)	OR	*p*
NAFLD patients				
FLI <60 n (%)	165 (65.7)	86 (34.3)	1.307 (0.924-1.848)	0.129
FLI ≥60 n (%)	46 (74.2)	16 (25.8)	1.959 (1.061-3.616)	0.030
NAFLD patients				
NFS <-1.455 n (%)	192 (68.3)	89 (31.7)	1.470 (1.047-2.064)	0.026
NFS ≥-1.455 n (%)	19 (59.4)	13 (40.6)	0.996 (0.474-2.090)	0.991
NAFLD patients				
FIB-4 <1.3 n (%)	177 (70.8)	73 (29.2)	1.652 (1.158-2.357)	0.005
FIB-4 ≥1.3 n (%)	34 (54.0)	29 (46.0)	0.799 (0.463-1.378)	0.419
Control	182 (59.5)	124 (40.5)	Reference	–

NFS, NAFLD fibrosis score; FIB-4, Fibrosis 4 Score; FLI, fatty liver index.

**Table 5:** Association between serum 25(OH)D_3_ levels and NAFLD.

Variable	NAFLD n (%)	Control n (%)	Univariate model	Adjusted model (OR (95%CI))		
				Model 1	Model 2	Model 3
Serum 25(OH)D_3_						
<12 ng/mL	51 (16.3)	52 (17.0)	Reference	Reference	Reference	Reference
12-20 ng/mL	160 (51.1)	130 (42.5)	1.255 (0.800, 1.969)	1.202 (0.763, 1.895)	0.562, (0.084,3.778)	0.154 (0.012,2.057)
>20 ng/mL	102 (32.6)	124 (40.5)	0.839 (0.526, 1.338)	0.740 (0.457, 1.198)	0.076 (0.006, 1.037)	0.019 (0.001, 0.619)
*p*_trend_	–	–	0.075	0.035	<0.001	<0.001

Model 1: Adjusted for age, gender. Model 2: Adjusted for age, gender, body mass index (BMI), insulin (INS), glucose (GLU). Model 3: Adjusted for age, gender, body mass index (BMI), insulin (INS), glucose (GLU), alanine aminotransferase (ALT), γ-glutamyl transpeptidase (GGT), alkaline phosphatase (ALP).

### 3.3 Vitamin D attenuates liver steatosis, serum lipid accumulation, and MCP–1 in NAFLD rats

**Figure 1:**
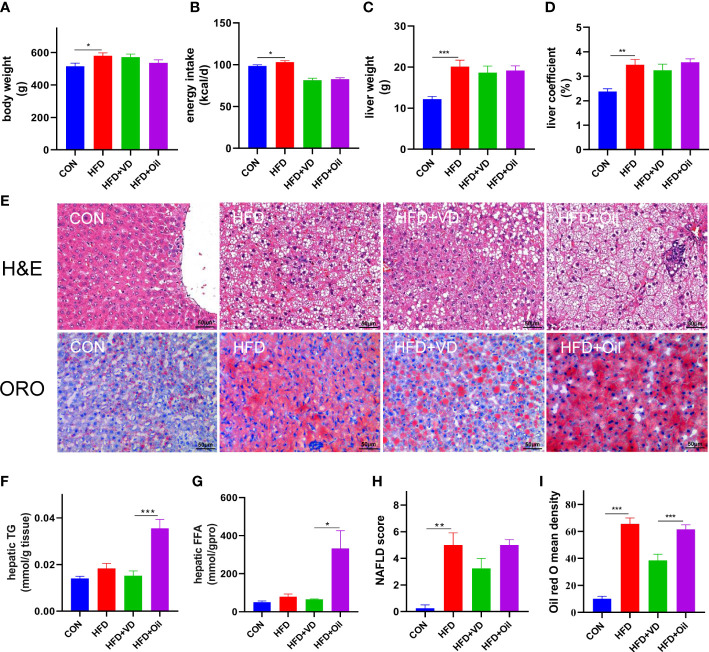
Vitamin D treatment ameliorate hepatic steatosis in NAFLD rats. **(A)** body weight, **(B)** energy intake, **(C)**liver weight, **(D)** liver coefficient, **(E)** H&E staining of liver tissues (400 × magnification) and Oil red O staining of liver tissue (400 × magnification). Four rats in each group were captured three views for evaluation of steatosis, lobular inflammation and balloon degeneration. Both scores are examined by an experienced pathologist who was unaware of the study. **(F)** Quantification of hepatic TG and **(G)** FFA in liver tissue. **(H)** NAFLD score and **(I)** Oil red O density of liver. Data were shown as the mean ± SEM. (n=8); ^*^P < 0.05, ^**^P < 0.01, ^***^P < 0.001.

**Figure 2:**
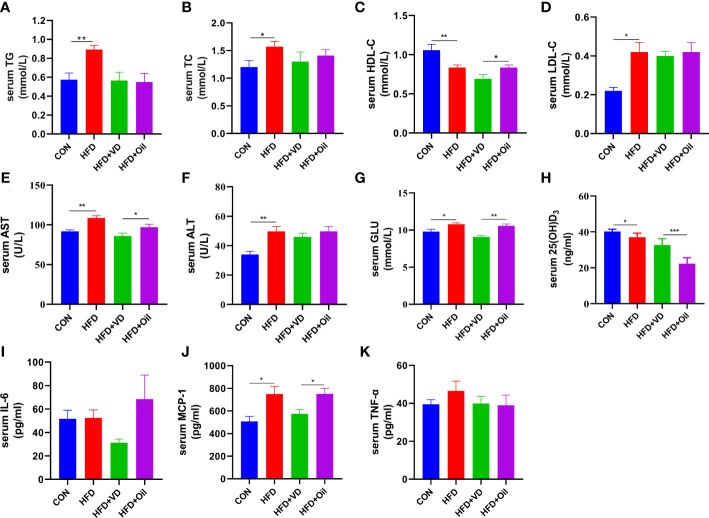
The effects of vitamin D in serum parameters of NAFLD rats. **(A)** serum TG, **(B)** TC, **(C)** HDL-C, **(D)** LDL-C, **(E)** AST, **(F)** ALT, **(G)** GLU, **(H)** 25(OH)D_3_, **(I)**IL-6, **(J)**MCP-1, **(K)**TNF-α levels. Data were shown as the mean ± SEM. (n=8) ^*^P < 0.05, ^**^P < 0.01, ^***^P < 0.001.

The body weight, energy intake, liver weight, and liver coefficient were significantly increased in HFD rats compared to those fed with a standard chow diet (**Figures 1A–D**). However, there was no significant improvement in body weight, energy intake, liver weight, or liver coefficient after VD treatment (**Figures 1A–D**). A large number of round transparent LDs were observed in the hepatic cells of HFD-induced rats with a small amount of ballooning degeneration and lobular inflammation, but these changes were ameliorated after VD treatment (**Figures 1E–G**). The HFD-induced elevated NAS and ORO mean density were also reduced after VD treatment (**Figures 1H, I**). Furthermore, the serum levels of TG, TC, LDL-C, AST, ALT, GLU, and MCP-1 were significantly increased after feeding rats a HFD diet, while the serum levels of HDL-C and 25(OH)D_3_ were significantly decreased after feeding rats a HFD. NAFLD rats treated with VD had significantly reduced serum levels AST, GLU, HDL-C, and MCP-1 but increased levels of 25(OH)D_3_; in addition, IL-6 showed a downward trend in HFD + VD rats compared to HFD + Oil rats (**Figures 2A–K**).

### 3.4 MK886 antagonizes VD-induced expression of β-oxidation-related proteins *in vivo*

**Figure 3:**
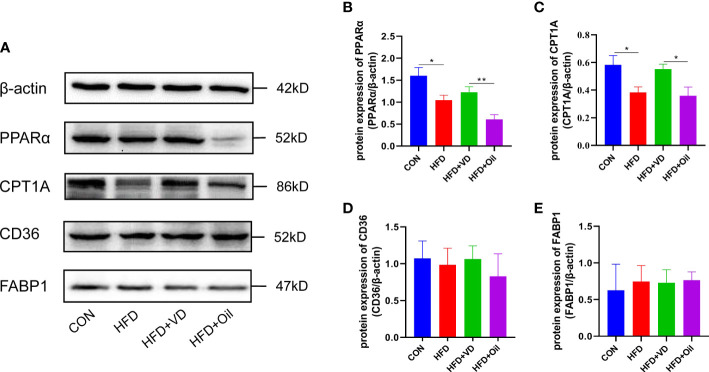
The effects of vitamin D on protein expression of lipid metabolism in NAFLD rats. **(A)** The expression of transcription factors involved in β-oxidation were detected by western blot. **(B)** PPARα and **(C)** CPT1A were qualified by β-actin. Lipid uptake proteins were detected by western blot. **(D)** CD36 and **(E)** FABP1 were qualified by β-actin. The band intensity ratios were analyzed by ImageJ. Data were shown as the mean ± SEM. (n=8) *P < 0.05, **P < 0.01.

**Figure 4:**
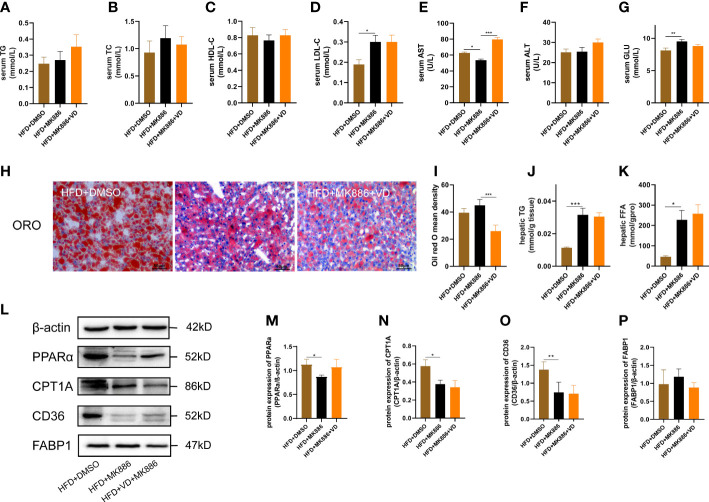
MK886 inhibited the effect of Vitamin D3. **(A)** serum TG, **(B)** TC, **(C)** HDL-C, **(D)** LDL-C, **(E)** AST, **(F)** ALT, **(G)** GLU, **(H)** Oil red O staining of liver tissue (400 × magnification), **(I)** Oil red O mean density, **(J)** hepatic TG, and **(K)** FFA, **(M–P)** the fold change in expression of the proteins in **(L)** relative to the expression of b-actin. *P < 0.05, **P < 0.01, ***P < 0.001.

The expression levels of hepatic lipid metabolism-associated proteins were measured by western blot analysis. The expression levels of PPARα and CPT1A were decreased in liver tissue in the HFD group (**Figures 3A–C**). After VD treatment, the expression levels of PPARα and CPT1A were increased (**Figures 3A–C**). The expression of CD36 and FABP1 showed no significant difference among the four groups (**Figures 3A, D, E**). Treatment with MK886, a PPARα antagonist, was used to explore the effect of VD on the PPARα signaling pathway. Compared to the HFD + DMSO group, serum LDL-C, serum GLU, hepatic TG, and hepatic FFA were decreased in the HFD + MK886 group, but serum AST was increased in the HFD + MK886 group; however, serum TG, TC, HDL-C, ALT, and ORO density were not significantly changed in the HFD + MK886 group (**Figures 4A–K**). VD treatment did not influence these indicators, except for serum AST and ORO density (**Figures 4E, I**). The expression levels of PPARα, CPT1A, and CD36 were decreased in the HFD + MK886 group compared to the HFD + DMSO group. However, VD treatment did not reverse the low expression of PPARα, CPT1A, and CD36 caused by MK886. Therefore, the effect of VD on reducing lipid uptake and elevating β-oxidation was suppressed by PPARα antagonism *in vivo* (**Figures 4L–P**).

### 3.5 Calcitriol treatment inhibits LD accumulation in HepG2 cells

**Figure 5:**
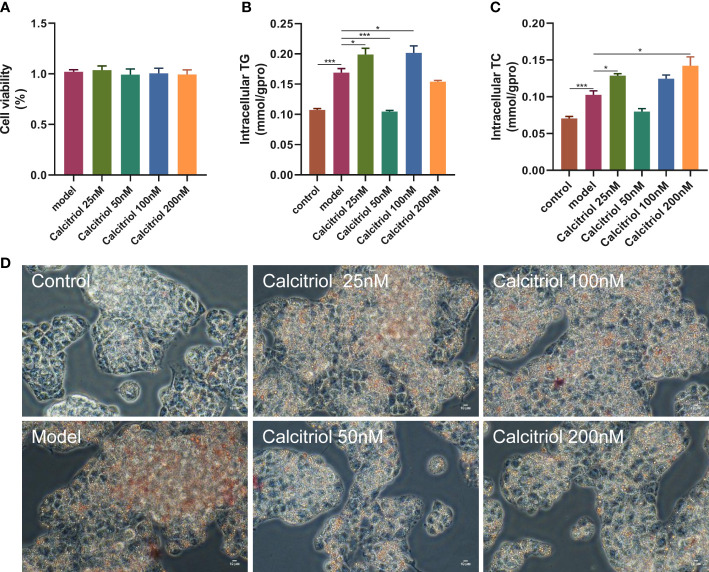
Effect of calcitriol on lipid deposition in HepG2 cells. Cytotoxic effects after **(A)** calcitriol (0-200nM) for 24 h were evaluated by CCK8 assay. **(B)** Intracellular TG and **(C)** TC were assayed in HepG2. **(D)** Intracellular lipid accumulation was detected by Oil red O staining method using an inverted microscope (400× magnification). Data were shown as the mean ± SEM. ^*^P < 0.05, ^***^P < 0.001.

To further confirm the beneficial effects of VD on hepatic steatosis, we established an *in vitro* model. Treatment with four concentrations (25, 50, 100, and 200 nM) of calcitriol did not affect the viability of HepG2 cells (**Figure 5A**). Moreover, LD accumulation, intracellular TG, and intracellular TC in OA-induced HepG2 cells were increased compared to control HepG2 cells (**Figures 5B–D**). Interestingly, treatment with 50 nM calcitriol significantly suppressed OA-induced lipid accumulation (**Figures 5B–D**).

### 3.6 MK886 reverses the amelioration of calcitriol in HepG2 cells

**Figure 6:**
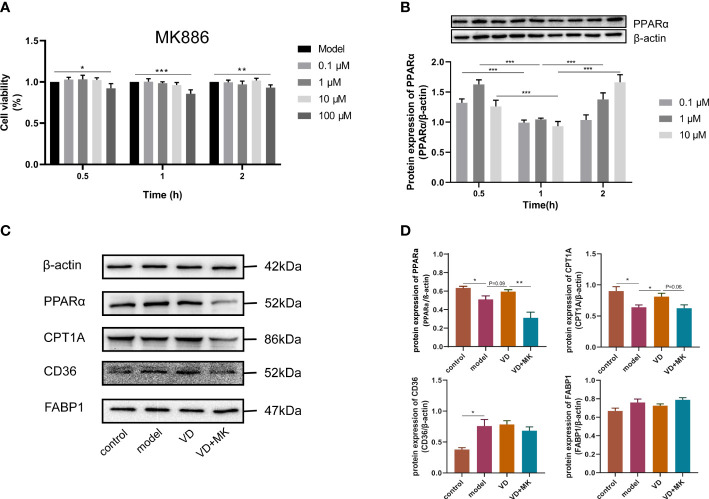
MK886 restrain the effect of VD treatment. **(A)** The cytotoxic effect after MK886 (0-100μM) for 0-2h was evaluated by CCK8 assay. **(B)** The best inhibiting effect after MK886 (0-10μM) for 0-2h was determined by western blot. **(C, D)** Effect of VD and MK886 on the protein expression involved in fatty acid uptake and β-oxidation, including PPARα, CPT1A, FABP1, CD36. The band intensity ratios were analyzed by ImageJ. Data are shown as means ± SEM. ^*^P < 0.05, ^**^P < 0.01, ^***^P < 0.001.

To determine the cytotoxic effect of MK886, the effects of different concentrations (0–100 μM) of MK886 for different lengths of time (0–2 h) on HepG2 cells were detected by the CCK8 assay. The cell viability was affected by 100 μM MK886 at 0.5, 1, and 2 h. Therefore, three doses of MK886 (0.1, 1, and 10 μM) were selected for treatment in subsequent experiments (**Figure 6A**). The protein expression of PPARα in model cells treated with different doses of MK886 for different times indicated that the optimal inhibitory effect occurred after treatment with 10 μM MK886 for 1 h (**Figure 6B**). Compared to control cells, the expression levels of PPARα and CPT1A were reduced in model cells, but the expression level of CD36 was elevated in model cells. After VD treatment of HepG2 cells, the protein expression of PPARα showed an elevated tendency, and the protein expression of CPT1A was significantly increased. In addition, MK886 decreased the protein expression levels of PPARα and CPT1A in the presence of VD *in vitro*, whereas the protein expression levels of CD36 and FABP1 were not significantly changed after treatment with both VD and MK886 (**Figures 6C, D**).

## 4 Discussion

In the present study, a case-control study was performed, which demonstrated that the prevalence of hyperlipidemia, liver dysfunction, diabetes, and obesity was higher in NAFLD patients than in controls even when stratified by gender. In addition, the serum ALT was negatively associated with 25(OH)D_3_ in NAFLD patients. In the early stage of fatty liver, low serum 25(OH)D_3_ concentration was associated with an increased risk of NAFLD. Adequate vitamin D state (25(OH)D_3_ >20 ng/mL) exhibited protective effects in NAFLD. VD treatment in rats and HepG2 cells ameliorated hepatic steatosis and systematic inflammation. In addition, VD did not exert an anti-steatosis effect *via* the regulation of lipid uptake and β-oxidation in the presence of the MK886 PPARα inhibitor. Therefore, these findings suggested that VD may exert its therapeutic effect on NAFLD by reducing lipid uptake and elevating β-oxidation *via* the PPARα signaling pathway.

VD deficiency is a global health problem regardless of the development index and latitude of the country. Liu et al. (48) reported that 20.7% of adults are VD deficient and that 63.2% of adults have VD inadequacy in the mainland of China. Furthermore, a low VD level has been identified as an independent risk factor for NAFLD (65). In general, the prevalence of NAFLD is higher in men than women, and it is higher in postmenopausal women than premenopausal women, which is attributed to the hepatic protective effect of estradiol (66, 67). Thus, we adjusted the effect of age and gender by matching and multiple logistic regression. In the present study, the NAFLD patients had higher BMI, WC, SBP, DBP, hepatic dysfunction, glucose metabolic disorder, and lipid metabolic disorder with a high prevalence of diabetes, hypertension, overweight, and obesity, which are related to metabolic syndromes (68–70). Previous studies reported lower serum 25(OH)D_3_ in Europeans and patients from southeast China than in controls (53, 55). A previous meta-analysis based on a large population reported an inverse correlation between serum 25(OH)D_3_ and NAFLD in European individuals (71). The present study showed that serum 25(OH)D_3_ was not significantly lower in NAFLD patients from southwest China, which may due to the BMI differences between the NAFLD patients and controls. VD inadequacy is often observed in obese individuals, which can be attributed to VD sequestration in AT, resulting in decreased availability, and diluted volumetrics due to larger AT mass (72). However, the relationship between adipose storage of VD and serum 25(OH)D_3_ levels in obese individuals is complex and has not been completely elucidated (73).

Consistent with a previous meta-analysis, which reported that VD supplementation decreases serum ALT (42), the present study demonstrated that 25(OH)D_3_ was negatively correlated with circulating ALT in patients. To minimize the effect of BMI, we considered BMI as a covariate for multiple logistic regression analysis, aiming to reduce its confounding effect. The multiple logistic regression analysis indicated that a high level of serum 25(OH)D_3_ (>20 ng/mL) may be a protective factor of NAFLD, which was consistent with previous studies reporting that adequate VD level plays a protective role by improving glucose metabolism and reducing inflammatory factors (43, 74, 75). In the present study, low VD status was a risk factor for NAFLD patients with steatosis and no advanced fibrosis (FLI≥60 or NFS<-1.455 or FIB-4<1.3). However, Xiu et al. (76) reported that serum VD levels in patients with hepatic fibrosis decreases with fibrosis progression. These differences may be attributed to several reasons. First, there were different sources of patients. Patients in the present study were from the Health Management Centre, in which patients typically present in the early stage of fatty liver disease without liver fibrosis, whereas Xiu et al. included patients with liver fibrosis presented to the Department of Endocrinology. Second, the study types were different. The study by Xiu et al. was a cross-sectional study that recruited patients with T2DM and divided them into two groups, namely, the T2DM with NAFLD group and T2DM without NAFLD group, whereas the present study used a case-control study with two groups, namely NAFLD patients and controls. Third, both studies identified alcohol abuse differently. In the present study, alcohol abuse was defined as <140 g/week for women and <210 g/week for men based on guidelines of NAFLD in China (47), while Xiu et al. excluded ethanol intake of <70 g/week for women and <140 g/week in men. Fourth, the relationship between VD and NAFLD may be influenced by VD-related gene variation (77). Finally, BMI may act as a confounding factor.

Based on the results of the present case-control study, NAFLD rats and HepG2 cells were used to explore the mechanism of VD supplementation on NAFLD. HFD is a well-established modeling method, which can lead to the development of metabolic syndrome, hepatic steatosis, and NASH in experimental animals (78). The duration of dietary intake and VD treatment in previous studies ranges from 10 weeks to 22 weeks and 3 weeks to 10 weeks, respectively, thus impacting the severity of NAFLD and effect of treatment (56, 79–81). In the present study, rats were fed with an HFD for 14 weeks, and the NAFLD model was determined by sacrifice at Week 7. Recent studies reported that VD ameliorates hepatic steatosis induced by a HFD and improves NAFLD in animal models (82, 83). However, the pathway of VD therapy for NAFLD treatment in the animal model is unclear. The NAFLD model induced by a HFD simulates the symptoms of human NAFLD with the accumulation of serum TG, TC, LDL-C, AST, ALT, GLU, and MCP-1 as well as hepatic steatosis. Similar to previous studies, the HFD-induced NAFLD model demonstrated higher body weight, liver weight, and liver index, which coincided with hyperlipidemia, hepatic dysfunction, hyperglycemia, and lower 25(OH)D_3_ (84–86). The hepatic tissue in the HFD group showed lipid vacuoles and ballooning degeneration. In these rats, VD supplementation did not influence the body weight or liver weight, which may be due to the inability of single nutrients to improve the disease phenotype. However, hepatic function, hepatic steatosis, systematic inflammation, hyperglycemia, and 25(OH)D_3_ were ameliorated by VD treatment as reported in previous studies (84–86). Exposure of HepG2 cells to 0.5 mM OA has been demonstrated to evoke hepatic steatosis, and this concentration was used in the present study (64). Similarly, OA-induced HepG2 cells showed intracellular lipid accumulation. Quantitative analyses of intracellular TG and LDs indicated that treatment with 50 nM calcitriol improved steatosis and resulted in fewer LDs. Li et al. (87) demonstrated that treatment with 100 nM calcitriol protects against hepatic steatosis by inducing autophagy. The differences in these studies may be due to focusing on different pathways. The benefits of VD supplementation were reflected in indicators rather than phenotype in accordance with clinical trials as in previous studies, suggesting that VD is an effective strategy for repressing NAFLD (88, 89).

**Figure 7:**
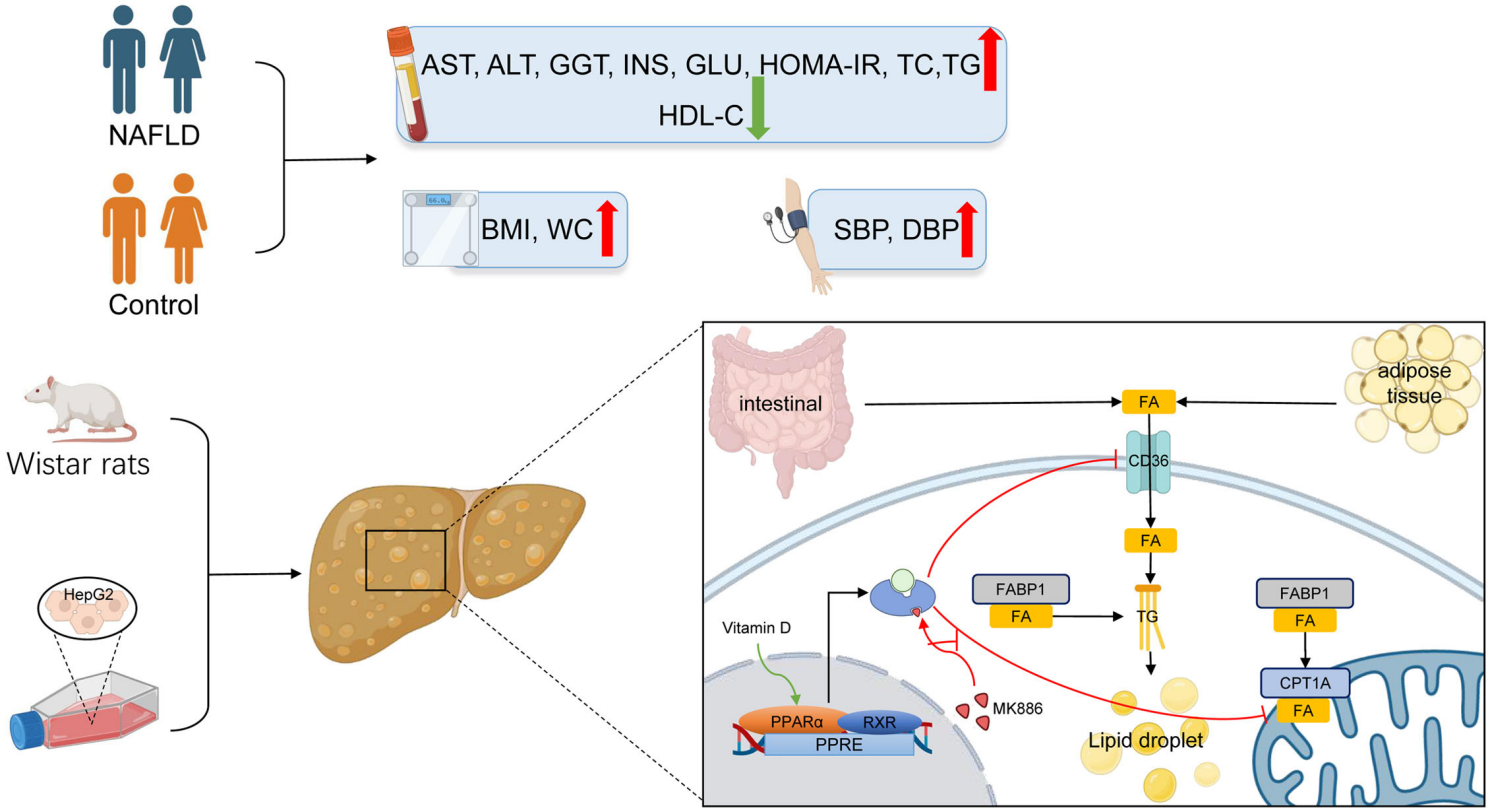
The lipid mechanism of NAFLD and anti-lipid accumulation mechanism of VD associated with PPARa pathway. Patients with NAFLD exhibited higher BMI, WC, SBP, DBP, AST, ALT, GGT, ALP, INS, GLU, HOMA-IR, TC, TG, LDL-C and lower HDL-C. The increased lipid oxidation and lipid uptake related proteins by VD treatment were inhibited after the application of MK886 in vivo and *in vitro*.

The PPARα pathway is an important sensing pathway for regulating lipid metabolism balance, and it may be an important target for the treatment of NAFLD *in vivo* and *in vitro* (90–93). In the present study, the protein expression levels of PPARα and CPT1A were decreased by HFD-induced steatosis, suggesting that reduced β-oxidation is one of the vital causes of steatosis. In contrast to a study by Yu et al. (94), CD36 and FABP1 failed to respond to HFD in the present study, which may be attributed to species differences. In accordance with a study by Refaat et al. (95), the present study demonstrated that VD increased the protein expression of PPARα in the liver. Accordingly, the protein expression of CPT1A was also increased after VD treatment. The protein expression levels of CD36 and FABP1 were not significantly changed after treatment with VD. Moreover, OA induced steatosis in HepG2 cells by decreasing lipid oxidation and increasing lipid uptake. After treatment with calcitriol, HepG2 cells in the model group showed improved intracellular steatosis, which was accompanied with higher protein expression levels of PPARα and CPT1A. However, the therapeutic effects of VD were inhibited by MK886 both in NAFLD rats and HepG2 cells. Therefore, these results indicated that VD treatment may regulate lipid uptake and β-oxidation *via* the PPARα signaling pathway (**Figure 7**).

The present study had several limitations. First, the present study only detected the expression of proteins related to lipid uptake and β-oxidation. Proteins related to *de novo* lipogenesis and lipid secretion should be explored in further studies. Second, relevant results were observed at the protein level but not at the gene level. Last, multiple doses of VD evaluated *in vivo* will increase the credibility of our hypothesis in future studies. Therefore, it remains to be explored whether vitamin D is a mediator or cause of NAFLD progression. In addition, long-term, multicenter RCTs that are controlled for confounders, including light exposure, with large sample sizes are still needed to detect the therapeutic effect of VD supplementation on NAFLD in the future.

In conclusion, the present study indicated that a high serum level of 25(OH)D_3_ may act as a protective factor in NAFLD patients. In addition, VD supplementation may alleviate hepatic steatosis by regulating lipid uptake and β-oxidation *via* the PPARα signaling pathway.

## Data availability statement

The original contributions presented in the study are included in the article/**Supplementary Materials**. Further inquiries can be directed to the corresponding authors.

## Ethics statement

The studies involving human participants were reviewed and approved by the ethic research committee of the Affiliated Hospital of Southwest Medical University. The patients/participants provided their written informed consent to participate in this study. The animal study was reviewed and approved by the ethic research committee of the Affiliated Hospital of Southwest Medical University.

## Author contributions

Study conception and design: JZ, YZ, and LM. Acquisition of data and analysis and interpretation of data: TD, LX, JZ, CY, WZ, and JL. Drafting the article: TD, YZ, and LM. Final approval of the version of the article to be published: all authors, and that all authors agree to be accountable for all aspects of the work.
