# Supplementary material for: Vitamin D improves hepatic steatosis in NAFLD via regulation of fatty acid uptake and β-oxidation
Source: Front Endocrinol (Lausanne). 2023 Mar 22;14:1138078. doi: 10.3389/fendo.2023.1138078 (PMC10074590; doi:10.3389/fendo.2023.1138078)
Supplement: Supplementary file 1 [file Table_1.docx]

Supplementary table 1 Composition of the experimental feed per 100g

| Nutrients | Chow diet | High-fat diet |
| --- | --- | --- |
| Total protein (g(kcal%)) | 21.1 (19.7) | 24.2 (19.8) |
| Carbohydrate (g(kcal%)) | 60.6 (70.3) | 40.1 (31.7) |
| fat (g(kcal%)) | 4.5 (10.0) | 27.4 (48.5) |
| Crude fiber (g) | 7.7 | 5.8 |
| Lysine (g) | 1.8 | 1.7 |
| Methionine + Cystine (g) | 0.9 | 0.8 |
| Arginine (g) | 1.5 | 1.3 |
| Histidine (g) | 0.8 | 0.9 |
| Tryptophan (g) | 0.3 | 0.4 |
| Phenylalanine (g) | 1.6 | 1.6 |
| Threonine (g) | 0.6 | 0.6 |
| Leucine (g) | 1.7 | 1.6 |
| Isoleucine (g) | 1.4 | 1.4 |
| Valine (g) | 1.7 | 1.6 |
| Calcium (g) | 1.8 | 1.7 |
| Phosphorus (g) | 1.2 | 1.1 |
| Magnesium (g) | 0.23 | 0.23 |
| Potassium (g) | 0.4 | 0.4 |
| Sodium (g) | 0.2 | 0.1 |
| Iron (mg) | 13.3 | 13.2 |
| Manganese (mg) | 8.7 | 8.5 |
| Copper (mg) | 1.4 | 1.4 |
| Zinc (mg) | 3.0 | 3.1 |
| Vitamin A (IU) | 1300 | 1300 |
| Vitamin D (IU) | 180 | 180 |
| Vitamin E (IU) | 14 | 14 |
| Vitamin K (IU) | 0.7 | 0.7 |
| kcal/g | 3.6 | 4.7 |

IU, international unit.
